# Towards Dynamic V2X Infrastructure: Joint Deployment and Optimization of 6DMA-Enabled RSUs

**DOI:** 10.3390/s26020388

**Published:** 2026-01-07

**Authors:** Xianjing Wu, Ruizhe Huang, Chuliang Wei, Xutao Chu, Junbin Chen, Shengjie Zhao

**Affiliations:** 1 Department of Electronic Engineering, College of Engineering, Shantou University, Shantou 515063, China; xianjingwu@stu.edu.cn (X.W.); 23rzhuang@stu.edu.cn (R.H.); junbinchen@stu.edu.cn (J.C.); 2 School of Computer Science and Engineering (School of Artificial Intelligence), Chongqing University of Science and Technology, Chongqing 401331, China; xutao_chu@cqust.edu.cn; 3 School of Computer Science and Technology, Tongji University, Shanghai 201804, China; shengjiezhao@tongji.edu.cn

**Keywords:** vehicle-to-everything (V2X), 6DMA, roadside unit (RSU) deployment, spatio-temporal analysis

## Abstract

The evolution towards 6G is set to transform Vehicle-to-Everything (V2X) networks by introducing advanced technologies such as Six-Dimensional Movable Antenna (6DMA). This technology endows Roadside Units (RSUs) with dynamic beam-steering capabilities, enabling adaptive coverage. However, traditional RSU deployment strategies, optimized for static coverage, are fundamentally mismatched with these new dynamic capabilities, leading to a critical deployment–optimization mismatch. This paper addresses this challenge by proposing **DyDO**, a novel **Dy**namic **D**eployment and **O**ptimization framework for the utilization of 6DMA-RSUs. Our framework strategically decouples the problem into two modules operating on distinct timescales. On a slow timescale, an offline deployment module analyzes long-term historical traffic data to identify optimal RSU locations. This is guided by a newly proposed metric, the Dynamic Potential Score (DPS), which quantifies a location’s intrinsic value for dynamic adaptation by integrating spatial concentration, temporal volatility, and traffic magnitude. On a fast timescale, an online control module employs an efficient Sequential Angular Search (SAS) algorithm to perform real-time, adaptive beam steering based on immediate traffic patterns. Extensive experiments on a large-scale, real-world trajectory dataset demonstrate that **DyDO** outperforms conventional static deployment methodologies. This work highlights the necessity of dynamic-aware deployment to fully unlock the potential of 6DMA in future V2X systems.

## 1. Introduction

Vehicle-to-Everything (V2X) communication, a cornerstone of future Intelligent Transportation Systems (ITS), is pivotal for enhancing road safety, traffic efficiency, and autonomous driving capabilities [1]. However, the practical realization of these benefits is challenged by the inherent complexities of vehicular environments. Urban scenarios are characterized by complex propagation conditions, frequent signal obstructions, and extreme dynamics due to high vehicle mobility and fluctuating densities [2]. These factors severely compromise the reliability and efficiency of conventional communication systems. As critical V2X infrastructure components, traditional Roadside Units (RSUs) are often deployed based on static, omnidirectional, or sectorized coverage models [3]. These models are ill-suited to the volatile spatio-temporal distribution of vehicular users, frequently resulting in coverage gaps and inefficient resource utilization.

**Figure 1:**
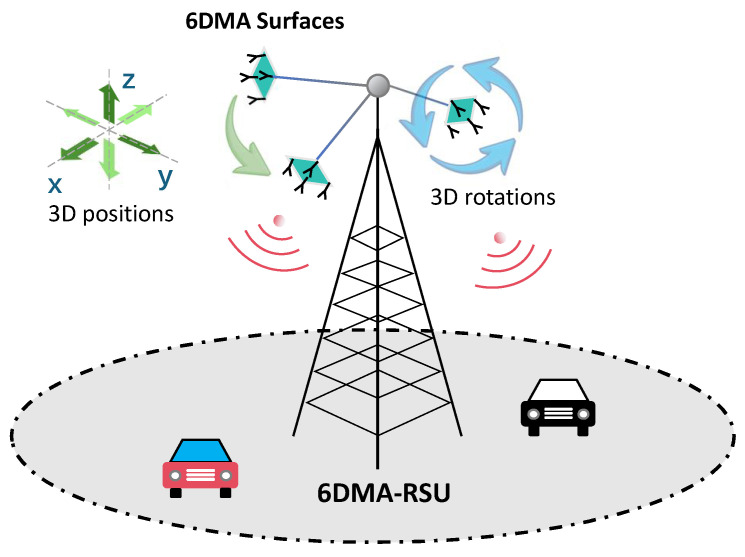
An illustrative scenario of 6DMA-RSUs dynamically serving vehicular traffic.

The evolution towards 6G introduces transformative technologies to address these limitations. Among them, Six-Dimensional Movable Antenna (6DMA)—an innovative antenna paradigm enabling independent adjustment in 3D position and 3D rotation (i.e., six degrees of freedom, 6DoF)—presents a groundbreaking solution [4], as conceptually illustrated in Figure 1. The principal advantage of 6DMA lies in its capacity for physical reconfiguration. By dynamically repositioning and reorienting the antenna array, a 6DMA system can proactively seek favorable channel conditions, circumvent obstructions, track high-mobility vehicles, and focus energy on transient traffic hotspots [5]. Integrating this technology transforms conventional RSUs into 6DMA-RSUs, marking a paradigm shift from static infrastructure provisioning to dynamic, adaptive service delivery.

Despite its promise, the integration of 6DMA introduces new, profound challenges beyond antenna-level optimization. Current research predominantly focuses on optimizing the 6DoF parameters for a fixed base station location [6], overlooking a critical constraint: the fixed physical deployment of an RSU fundamentally bounds the performance gains achievable through dynamic parameter tuning. Simply replacing static antennas with movable ones is insufficient. While 6DMA grants unprecedented flexibility, it also creates a more intricate optimization landscape. This leads to the core research question addressed in this work: *How can the full potential of 6DMA-RSUs be unleashed within complex V2X environments?* We decompose this overarching problem into two interdependent sub-problems operating on distinct timescales: (1) Macroscopic/Slow-Timescale Problem (Strategic deployment): Where should 6DMA-RSUs be deployed within the urban infrastructure? A suboptimal deployment can critically constrain the benefits of subsequent dynamic tuning, leading to a scenario of stranded potential, where advanced capabilities remain underutilized. (2) Microscopic/Fast-Timescale Problem (Real-time Control): Given a fixed deployment location, how should the 6DMA antenna parameters (specifically, beam orientations) be adjusted in real-time based on instantaneous traffic conditions to maximize coverage performance?

To tackle this dual challenge, this paper proposes **DyDO**, a novel **Dy**namic **D**eployment and **O**ptimization framework for 6DMA-RSUs’ better utilization. Our framework systematically addresses both timescales:

- Stage 1 (Strategic deployment—Slow Timescale): In the offline planning phase, the framework leverages historical traffic data (e.g., Global Positioning System (GPS) trajectories) to perform “deployment for optimal adaptability.” The objective is not merely to satisfy basic coverage but to identify locations with the highest strategic potential to maximize the “maneuver value” of 6DMA. This is achieved through a novel metric, the Dynamic Potential Score (DPS), and a greedy deployment algorithm.
- Stage 2 (Real-time Control—Fast Timescale): During online operation, each deployed 6DMA-RSU employs an efficient algorithm, Sequential Angular Search (SAS), to continuously adapt its beam orientations based on real-time (or near-real-time) traffic information, thereby maximizing instantaneous network coverage.

The primary contributions of this work are summarized as follows:

1. Novel Dual-Timescale Framework for Dynamic V2X Infrastructure: To the best of our knowledge, this is the first framework that jointly addresses the strategic deployment and tactical control problems for dynamic-capability RSUs like 6DMA. By explicitly decoupling decisions across slow (deployment) and fast (control) timescales, our framework resolves the inherent “deployment-control mismatch” overlooked by prior studies.
2. Dynamic Potential Score (DPS) for Strategic Deployment: We introduce the DPS, a novel data-driven metric that fundamentally departs from traditional average-coverage criteria. The DPS quantifies a location’s intrinsic value for dynamic adaptation by integrating spatial traffic concentration, temporal volatility, and baseline traffic magnitude, enabling a paradigm shift towards “dynamic-gain-aware” deployment.
3. Efficient Real-Time Control Algorithm and Rigorous Validation: We develop the Sequential Angular Search (SAS) algorithm, a low-complexity heuristic (O(M×L)) that enables practical real-time beam steering for multi-beam 6DMA systems. We validate the entire framework through an extensive comparative study (Static-Optimal vs. Predictive-Dynamic vs. Oracle-Dynamic) on a large-scale real-world dataset, providing a quantification of the potential of dynamic deployment.

## 2. Related Work

This section reviews the literature in three key areas relevant to our work: conventional RSU deployment strategies, emerging 6G networking technologies, and dynamic network control methodologies. We identify the limitations of prior art, which collectively motivate the novel dual-timescale approach proposed in this paper.

### 2.1. Conventional RSU Deployment Strategies

The problem of RSU deployment has been extensively studied for traditional V2X systems, with strategies primarily focusing on static optimization objectives [7,8].

A significant body of research has pursued coverage-centric objectives, aiming to maximize geographic road coverage or the volume of served vehicular traffic [9]. Methodologies range from greedy algorithms that iteratively select locations offering the maximum marginal coverage gain [10,11], to more complex heuristic and meta-heuristic approaches such as genetic algorithms and particle swarm optimization [12]. Another prevalent strategy involves intersection-based deployment, which prioritizes road junctions due to their natural traffic convergence, often utilizing centrality metrics to identify critical intersections [8,13]. Recent work, such as Greta [14], has further advanced this field by employing reinforcement learning to adapt to various metrics; however, it still fundamentally operates under a static coverage assumption.

A second category of research has focused on Quality-of-Service (QoS)-centric objectives, considering metrics like communication delay [8,15], throughput, and connectivity. These studies typically formulate complex optimization problems to place RSUs in a manner that guarantees specific performance levels for V2X applications.

A critical limitation common to all these conventional approaches is their foundation on a static coverage model, where an RSU’s service area is assumed to be a fixed, typically circular or sectorized region. This fundamental assumption is incompatible with the dynamic, steerable beam capabilities of 6DMA-RSUs. Consequently, these existing deployment strategies are inherently ill-suited to identify locations that can fully leverage the adaptive nature of next-generation V2X infrastructure.

### 2.2. Emerging Technologies in 6G Networks

The advent of 6G has spurred research into new technologies designed to meet future communication demands. Two areas are particularly relevant to our work.

First, the deployment of Intelligent Reflecting Surfaces (IRS) presents a related infrastructure deployment problem [16]. Research in this domain focuses on optimizing the locations of passive IRS panels to intelligently reconfigure the wireless propagation environment [17,18], thereby enhancing signal strength and coverage [19]. While analogous as a deployment problem, the objective of IRS deployment is to assist existing communication links rather than to serve as a primary access point, and its control mechanisms are fundamentally different from those of active 6DMA systems [20].

Second, research into multi-dimensional movable antenna technologies for 6G has been prolific. Foundational studies on 6G outline the vision and key enabling technologies, including the transition towards more dynamic and intelligent radio environments [21]. Furthermore, specific techniques like Orbital Angular Momentum (OAM) have been explored to dramatically increase spectral efficiency and channel capacity [22,23]. However, the overwhelming focus of this research has been confined to the physical and link layers, emphasizing signal processing, channel modeling, and performance analysis [24]. The crucial, higher-level problem of how to strategically deploy infrastructure that is endowed with such dynamic physical-layer capabilities, particularly considering the interplay between placement and optimization for 6DMA, remains a largely uncharted territory.

### 2.3. Dynamic Network Control

The challenge of dynamic control in networked systems has been addressed using various techniques. Reinforcement Learning (RL), in particular, has demonstrated considerable promise in domains such as dynamic resource allocation [25,26] and UAV trajectory planning for aerial base stations [27,28], owing to its ability to adapt to changing conditions without explicit system models. However, these methods typically assume that the physical infrastructure (e.g., base station locations) is pre-determined and fixed. They effectively solve the tactical control problem but do not address the antecedent strategic problem of where to place the infrastructure to maximize the effectiveness of the subsequent control [29]. While RL could theoretically be applied to our fast-timescale beam steering problem, the specific structure of the multi-beam selection lends itself to a more direct and computationally efficient heuristic approach, such as our proposed SAS algorithm. This allows us to avoid the complexities associated with RL training and state representation design for this particular subproblem.

### 2.4. Positioning of This Work

Our work is situated at the confluence of these three research areas, and directly addresses the identified gaps. Unlike conventional RSU deployment strategies, our framework is explicitly designed for the dynamic nature of 6DMA-RSUs. In contrast to the predominant focus of 6G physical layer research, we elevate the perspective to the critical system-level problem of infrastructure deployment. Furthermore, diverging from typical dynamic control studies that assume fixed sites, we jointly optimize both deployment and control. This is achieved through a novel metric (DPS) for strategic deployment and an efficient heuristic (SAS) for real-time control. This holistic, dual-timescale approach is specifically tailored to address the unique challenges and opportunities presented by next-generation dynamic V2X infrastructure.

## 3. System Model

This section establishes the foundational models for the network environment, the 6DMA-RSU capabilities, and formally defines the joint deployment and control optimization problem.

### 3.1. Network Scenario and Vehicle Traffic Model

We model the operational environment based on a real-world urban setting. The geographical area of interest is discretized into a uniform grid within a two-dimensional coordinate system. Specifically, we utilize the Universal Transverse Mercator (UTM) projection to map raw GPS coordinates (latitude, longitude) to metric coordinates (x,y). The area is partitioned into a grid of cells, indexed by (i,j), each representing a square region of size G×G meters (e.g., G=25 m).

In this V2X scenario, the network is designed to support the transmission of diverse information signals critical for intelligent transportation. These signals primarily include high-bandwidth downlink services, such as High-Definition (HD) map updates and collective perception messages (sharing sensor data like LiDAR point clouds), as well as reliability-critical control signaling for autonomous driving coordination. The 6DMA-RSUs aim to establish robust communication links to deliver these signals effectively. Consequently, ensuring geometric coverage for vehicles (represented by GPS points) serves as a prerequisite for satisfying the Quality of Service (QoS) requirements of these information flows.

Vehicular traffic distribution and mobility patterns are derived from a large-scale GPS trajectory dataset. The raw GPS points p=(lon,lat,timestamp) undergo the following preprocessing pipeline:

1. Coordinate Conversion: Raw coordinates are converted to UTM (x,y).
2. Temporal Discretization: The operational period *T* (e.g., 24 h) is divided into uniform timeslots of duration $T_{k}$ (e.g., 5 min), indexed by k∈{1,…,K}, where $K=T/T_{k}$.
3. Spatio-Temporal Aggregation: Each GPS point *p* occurring in timeslot *k* is mapped to its corresponding grid cell (i,j).

The aggregated traffic data is encapsulated in a 3D data cube *C*, where C[i,j,k] denotes the total count of GPS points within grid cell (i,j) during timeslot *k*. Let P(k) represent the set of all GPS points occurring in timeslot *k*.

### 3.2. 6DMA-RSU Coverage Model

**Figure 2:**
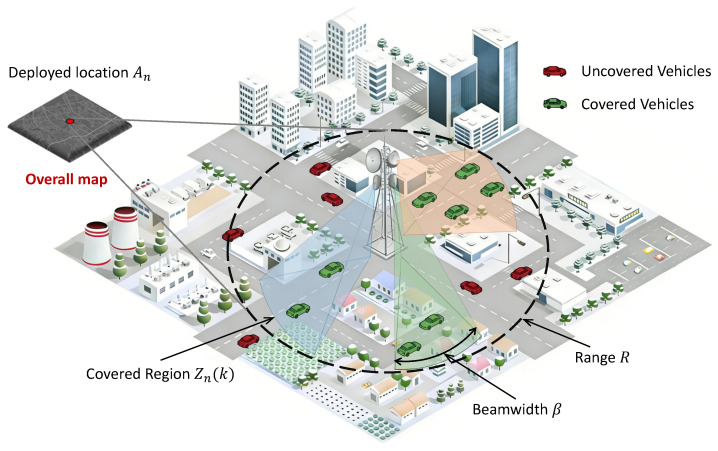
The proposed V2X scenario with 6DMA-RSUs.

We abstract the physical-layer complexities of 6DMA into a tractable coverage model suitable for system-level optimization of V2X networks, as shown in Figure 2.

#### 3.2.1. Antenna Abstraction and Adjustable Parameters

Each 6DMA-RSU *n* is deployed at a fixed macro-location $A_{n}$, corresponding to a grid cell $\left(i_{n},j_{n}\right)$. We assume each RSU is equipped with an antenna array capable of generating *M* concurrent directional beams (M=3 by default). Each beam m∈{1,…,M} is characterized by a fixed beamwidth β (e.g., $60^{\circ }$) and an adjustable center angle (boresight) $\theta_{n,m}$. The RSU has a maximum communication range *R*.

The primary adjustable parameter for RSU *n* at timeslot *k* is the set of beam center angles, denoted as $\Theta_{n}\left(k\right)=\left\{\theta_{n,1}\left(k\right),\theta_{n,2}\left(k\right),\ldots ,\theta_{n,M}\left(k\right)\right\}$, where $\theta_{n,m}\left(k\right)\in \left[0^{\circ },360^{\circ }\right)$.

#### 3.2.2. Coverage Definition

The coverage region of RSU *n* at timeslot *k*, denoted $Z_{n}\left(k\right)=\mathrm{Coverage}\left(A_{n},\Theta_{n}\left(k\right)\right)$, is defined as the union of the *M* angular sectors. Each sector is defined by its center angle $\theta_{n,m}\left(k\right)$, beamwidth β, and bounded by the maximum range *R*.

In this study, we interpret each raw GPS point *p* as a discrete unit of traffic demand at a specific timestamp. Since a single vehicle generates a sequence of GPS points along its trajectory, covering a GPS point effectively signifies that the RSU provides connectivity to the corresponding vehicle during the time interval represented by that sample. Consequently, maximizing the total number of covered GPS points is equivalent to maximizing the total service duration or served traffic load of the V2X network.

A GPS point *p* with Cartesian coordinates $\left(x_{p},y_{p}\right)$ is considered covered by RSU *n* at timeslot *k* if and only if: 1. The Euclidean distance between *p* and $A_{n}$ satisfies $\mathrm{dist}(p,A_{n})\leq R$. 2. The azimuth angle $\phi (p,A_{n})$ of point *p* relative to $A_{n}$ lies within the angular span of at least one beam *m*, i.e., $\phi \left(p,A_{n}\right)\in \left[\theta_{n,m}\left(k\right)-\beta /2,\theta_{n,m}\left(k\right)+\beta /2\right]$ (angles handled modulo $360^{\circ }$).

Let $I_{n,k}\left(p\right)$ be the indicator function for this coverage event:

(1) $$I_{n,k}\left(p\right)=\left\{\begin{array}{cc} 1, & \mathrm{if}\;p\;\mathrm{is}\;\mathrm{covered}\;\mathrm{by}\;\mathrm{RSU}\;n\;\mathrm{at}\;\mathrm{timeslot}\;k,\\ 0, & \mathrm{otherwise}. \end{array}\right.$$

The set of points covered by RSU *n* in timeslot *k* is $C_{n}\left(k\right)=\left\{p\in P\left(k\right)|I_{n,k}\left(p\right)=1\right\}$.

It is worth noting that while the optimization framework focuses on the spatial beam alignment to maximize geometric coverage, we implicitly assume that valid physical-layer connectivity is supported by standard multiple access techniques. Specifically, when multiple GPS points (users) fall within the same directional beam of an RSU during a timeslot *k*, they are presumed to be served via orthogonal resource allocation schemes, such as Time Division Multiple Access (TDMA). This abstraction allows us to isolate and optimize the beam-level resource allocation problem.

### 3.3. Problem Formulation

#### 3.3.1. Overall Objective

The primary objective is to maximize the overall V2X network coverage performance over the entire operational period *T*. This is quantified as the total number of GPS points covered by the network of *N* deployed RSUs across all timeslots. Formally, we aim to solve

(2) $$\max_{A,\;{\left\{\Theta \left(k\right)\right\}}_{k=1}^{K}}\;C_{total}=\sum_{k=1}^{K}\left|\underset{n\in A}{⋃}C_{n}\left(k\right)\right|,$$

subject to

- A⊂P is the set of RSU deployment locations, with |A|=N.
- $\Theta \left(k\right)=\{\Theta_{n}\left(k\right)|n\in A\}$ is the collection of all RSU beam angle configurations at timeslot *k*.

#### 3.3.2. Challenges and Intractability

However, it is non-trivial to directly solve the above optimization formulation in Equation (2), because of the following fundamental challenges:

1. Mixed Decision Space: The problem involves a combinatorial choice of deployment locations A from a large candidate set P, coupled with a functional optimization over continuous, time-varying beam angles {Θ(k)}.
2. High Dimensionality: The search space is enormous. For *N* RSUs, *M* beams, *K* timeslots, and *L* discrete angle choices per beam, the number of possible angle configurations alone is ${\left(L^{M}\right)}^{N\times K}$, superimposed on the |P|N deployment combinations.
3. Temporal Dynamics: The optimal angles Θ(k) are contingent on the instantaneous traffic pattern P(k), which exhibits complex, non-stationary dynamics.
4. Tight Coupling: The effectiveness of the deployment A is intrinsically linked to the potential performance achievable through real-time control {Θ(k)}, and vice versa. This bidirectional dependency precludes independent optimization.

### 3.4. Dual-Timescale Problem Decomposition

Given the intractability of the monolithic joint problem, we propose a principled decoupling based on the inherent timescale separation between the decisions:

- Subproblem 1: Slow-Timescale Strategic deployment: This offline problem focuses on the one-time selection of optimal RSU locations $A^{*}$. The objective shifts from maximizing instantaneous coverage to maximizing the long-term potential for dynamic adaptation. This subproblem is solved by analyzing the entire historical data cube *C* to compute a novel Dynamic Potential Score (DPS) for candidate locations, as detailed in Section 4.
- Subproblem 2: Fast-Timescale Tactical Control: Given a fixed deployment $A^{*}$, this online problem involves determining the real-time beam angles $\Theta^{*}\left(k\right)$ for each timeslot *k* to maximize the instantaneous coverage $|\cup_{n\in A^{*}}C_{n}\left(k\right)|$. This subproblem is solved using an efficient, real-time algorithm (Sequential Angular Search) that reacts to recent traffic conditions, as described in Section 5.

This decomposition strategically addresses the problem’s complexity by applying tailored methodologies for each distinct timescale: leveraging long-term statistical patterns for robust infrastructure planning and short-term observations for agile operational control.

### 3.5. Framework Overview

**Figure 3:**
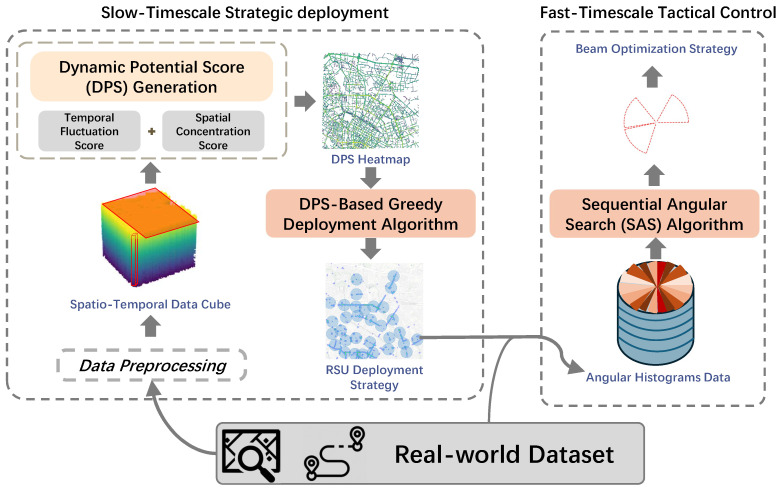
The Proposed Framework Architecture.

As shown in Figure 3, the **DyDO** framework addresses the complexity by separating decisions:

1. Slow Timescale (Offline Strategic Deployment): Using long-term historical data (calculated as a cube *C*), determine the fixed set of *N* deployment locations $A^{*}$ by optimizing a proxy metric (DPS) designed to reflect long-term dynamic potential. This is detailed in Section 4.
2. Fast Timescale (Online Tactical Control): Given the fixed locations $A^{*}$, determine the beam angles $\Theta^{*}\left(k\right)$ for each timeslot *k* based on recent or current traffic data. This uses the efficient SAS algorithm detailed in Section 5.

## 4. Slow-Timescale Strategic Deployment: A Potential-Driven Deployment Approach

This section details the first core module of **DyDO**: the offline, strategic selection of *N* RSU deployment locations. Departing from traditional coverage-centric metrics, we reformulate the deployment objective to prioritize locations that inherently offer the greatest potential for leveraging 6DMA’s adaptive beam-steering capabilities over the long term.

### 4.1. Dynamic Potential Score (DPS) Modeling

#### 4.1.1. Motivation: From Static Coverage to Dynamic Potential

Traditional RSU deployment strategies, such as maximum coverage algorithms, aim to maximize the number of unique users or road segments covered under a static antenna model (e.g., a fixed circular footprint). These approaches are fundamentally misaligned with 6DMA-RSUs, as they ignore the antenna’s ability to dynamically reconfigure its coverage pattern. A location deemed suboptimal for static coverage might be highly valuable if it allows a 6DMA-RSU to effectively track temporally shifting traffic hotspots through beam rotation. Conversely, deploying a sophisticated 6DMA-RSU in a location with static and uniform traffic patterns offers negligible advantage over a simpler static antenna. Therefore, the deployment objective must evolve from maximizing static coverage to maximizing the potential for dynamic coverage adaptation.

#### 4.1.2. Core Concept: The Dynamic Potential Score

We introduce the Dynamic Potential Score (DPS) as the core metric guiding our strategic deployment. The DPS of a candidate location (i,j) quantifies its intrinsic “maneuver value”—the degree to which deploying a 6DMA-RSU at this location is likely to yield significant performance gains through real-time beam steering compared to a static configuration. A high DPS indicates that the location experiences traffic patterns amenable to dynamic adaptation, enabling an RSU placed here to achieve superior long-term coverage by actively adjusting its beams.

#### 4.1.3. Quantifying DPS from Historical Data

The DPS is computed from the long-term historical traffic data cube *C*. It synthesizes two critical aspects of traffic dynamics: temporal fluctuation and spatial concentration, while also accounting for the baseline traffic volume. Formally, the DPS for a grid cell (i,j) is defined as

(3) $$\mathrm{DPS}\left(i,j\right)=S_{\mathrm{temporal}}\left(i,j\right)\times S_{\mathrm{spatial}}\left(i,j\right),$$

where $S_{\mathrm{temporal}}\left(i,j\right)$ is the temporal fluctuation score and $S_{\mathrm{spatial}}\left(i,j\right)$ is the spatial concentration score. These two components are detailed as follows.

Temporal Fluctuation Score ($S_{\mathrm{temporal}}$)

This component captures the volatility of traffic volume over time. A location with highly variable traffic (e.g., strong tidal flows during rush hours) benefits significantly from dynamic beam steering that can adapt to these fluctuations. We quantify this using the standard deviation of traffic counts across all timeslots:

(4) $$S_{\mathrm{temporal}}\left(i,j\right)=\sigma_{k}\left(C[i,j,k]\right),$$

where $\sigma_{k}$ denotes the standard deviation over the timeslot index *k*. A higher standard deviation indicates greater temporal volatility, contributing positively to the DPS.

Spatial Concentration Score ($S_{\mathrm{spatial}}$)

This component ensures that the traffic, besides being temporally volatile, is also spatially concentrated enough to be effectively served by directional beams. It is decomposed into a magnitude factor and a structural factor:

(5) $$S_{\mathrm{spatial}}\left(i,j\right)=S_{\mathrm{mag}}\left(i,j\right)\times {\bar{S}}_{\mathrm{rel}}\left(i,j\right).$$

The magnitude factor $S_{\mathrm{mag}}$ normalizes the mean traffic volume of the cell by the global maximum, ensuring a sufficient baseline of service demand:

(6) $$S_{\mathrm{mag}}\left(i,j\right)=\frac{\mu_{k}\left(C\left[i,j,k\right]\right)}{\max_{i^{\prime },j^{\prime },k^{\prime }}\left(C\left[i^{\prime },j^{\prime },k^{\prime }\right]\right)},$$

where $\mu_{k}$ denotes the mean over timeslots, and $\max_{i^{\prime },j^{\prime },k^{\prime }}C\left[i^{\prime },j^{\prime },k^{\prime }\right]$ represents the global maximum traffic count across the entire cube *C*, where the primed indices $\left(i^{\prime },j^{\prime },k^{\prime }\right)$ are used to iterate over all possible grid cells and timeslots in the spatio-temporal domain.

The relative structure score ${\bar{S}}_{\mathrm{rel}}$ measures, on average over time, the spatial clustering of traffic around the candidate cell. High values indicate that high traffic in the center cell is consistently accompanied by high traffic in its vicinity, forming a “hotspot” ideal for directional coverage. For each timeslot *k*, we compute a local similarity score $S_{{\mathrm{rel}}_{k}}\left(i,j\right)$ within a defined window (e.g., 9×9 cells centered on (i,j)). The similarity between the center cell $C_{k}=C\left[i,j,k\right]$ and a neighbor $N_{k}=C\left[i-m,j-n,k\right]$ is calculated using a normalized difference metric:

(7) $${\mathrm{Sim}}_{k}\left(C,N\right)=1-\frac{|\left(C_{k}+1\right)-\left(N_{k}+1\right)|}{\max(\left(C_{k}+1\right),\left(N_{k}+1\right))},$$

The addition of 1 mitigates the impact of zero counts. The timeslot-specific score is the Gaussian-weighted average of these similarities over the window:

(8) $$S_{{\mathrm{rel}}_{k}}\left(i,j\right)=\sum_{m,n\in \mathrm{Window}}W_{m,n}\cdot {\mathrm{Sim}}_{k}\left(C\left(i,j\right),C\left(i-m,j-n\right)\right),$$

where *W* is a 2D Gaussian kernel. The final relative structure score is the average over all timeslots:

(9) $${\bar{S}}_{\mathrm{rel}}\left(i,j\right)=\mu_{k}\left(S_{{\mathrm{rel}}_{k}}\left(i,j\right)\right).$$

**Figure 4:**
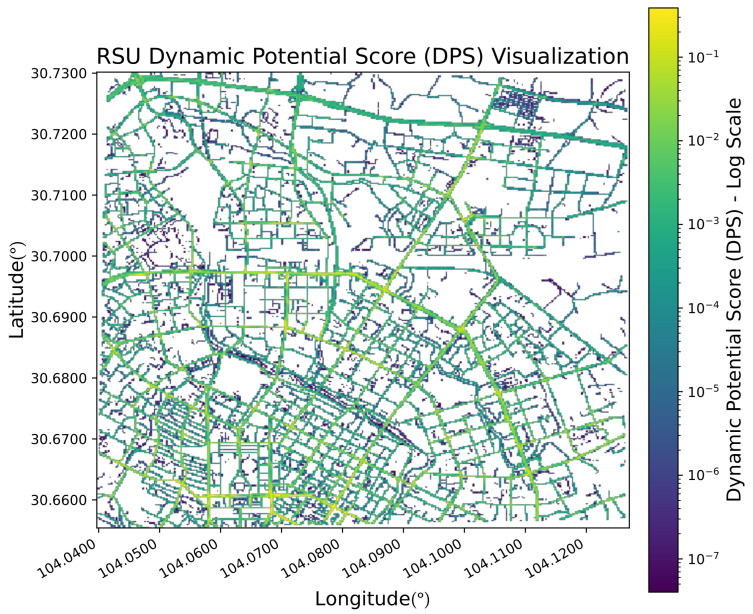
Heatmap of the Dynamic Potential Score (DPS) for central Chengdu. Brighter colors indicate higher potential for dynamic adaptation, characterized by high spatial concentration and temporal fluctuation of traffic.

The resulting DPS values form a heatmap (Figure 4) that highlights locations with high dynamic potential, guiding the subsequent deployment algorithm.

### 4.2. DPS-Based Greedy Deployment Algorithm

#### 4.2.1. Problem Formalization

Given the computed DPS heatmap *H* (where H[i,j]=DPS(i,j)), a deployment budget *N*, and the RSU coverage radius *R*, the strategic deployment problem is formulated as a maximum coverage problem. The objective is to select a set $A^{*}\subset P$ of *N* grid locations that maximizes the total DPS value covered by the network, accounting for potential overlaps:

(10) $$\max_{A\subset P,\;|A|=N}\sum_{\left(i,j\right)\in {⋃}_{A_{n}\in A}\mathrm{Cov}\left(A_{n}\right)}H\left[i,j\right],$$

Here, $\mathrm{Cov}(A_{n})$ denotes the set of grid cells covered by an RSU placed at $A_{n}$. This problem is known to be NP-hard.

#### 4.2.2. Algorithm Design

We employ a greedy heuristic algorithm as outlined in Algorithm 1 that iteratively selects the candidate location providing the highest marginal gain in total covered DPS. This approach effectively balances the selection of high-DPS locations with the minimization of coverage redundancy.
**Algorithm 1** Greedy Deployment based on DPS**Require:** DPS Heatmap *H*, Budget *N*, Coverage Radius $R_{\mathrm{grids}}$ (in grid units), Threshold ϵ**Ensure:** Optimal deployment locations $A^{*}$ (set of grid indices)   1: $A^{*}\leftarrow \emptyset$   2: $H_{\mathrm{current}}\leftarrow H.\mathrm{copy}\left(\right)$▷ Working copy of the heatmap   3: $K\leftarrow \mathrm{create}\_\mathrm{circular}\_\mathrm{kernel}(R_{\mathrm{grids}})$▷ 2D kernel representing coverage area   4: **for** k=1 to *N* **do**   5:    $S\leftarrow \mathrm{convolve}2d(H_{\mathrm{current}},K,\mathrm{mode}=‘\mathrm{same}’)$▷ Compute score for all candidates   6:    $\left(i^{*},j^{*}\right)\leftarrow \arg\max_{i,j}S\left[i,j\right]$▷ Find best candidate   7:    **if** $S[i^{*},j^{*}]<\epsilon$
**then**▷ Stop if marginal gain is negligible   8:          **break**   9:    **end if** 10:    $A^{*}\leftarrow A^{*}\cup \left\{\left(i^{*},j^{*}\right)\right\}$ 11:    $H_{\mathrm{current}}\leftarrow \mathrm{mask}\_\mathrm{area}(H_{\mathrm{current}},i^{*},j^{*},R_{\mathrm{grids}})$▷ Zero out covered area 12: **end for** 13: **return** $A^{*}$

The algorithm’s efficiency stems from using 2D convolution to simultaneously evaluate all candidate locations in each iteration. The circular kernel *K* approximates the coverage area of an RSU, and the mask_area function sets the DPS values within the covered region to zero, preventing redundant coverage in subsequent iterations. This greedy approach provides a computationally feasible and theoretically grounded approximation for the NP-hard deployment problem.

## 5. Fast-Timescale Tactical Control: Real-Time Beam Optimization

Given the strategically placed RSU locations $A^{*}$ determined offline, the fast-timescale module addresses the online operational challenge of real-time beam angle optimization. For each deployed 6DMA-RSU $n\in A^{*}$ and at every operational timeslot *k* (e.g., every 5 min), this module dynamically adjusts the *M* beam orientations $\Theta_{n}\left(k\right)$ to maximize the instantaneous coverage of vehicular traffic. This real-time adaptation is crucial for maximizing the effective service duration provided by the V2X network, requiring an algorithm capable of processing current traffic information and computing near-optimal beam configurations under stringent latency constraints.

### 5.1. Problem Formulation and Complexity Analysis

For a specific RSU *n* at timeslot *k*, the tactical control problem is to determine the optimal set of beam angles $\Theta_{n}\left(k\right)=\left\{\theta_{n,1}\left(k\right),\theta_{n,2}\left(k\right),\ldots ,\theta_{n,M}\left(k\right)\right\}$ that maximizes the number of GPS points covered within that timeslot:

(11) $$\max_{\Theta_{n}\left(k\right)}\left|C_{n}\left(k\right)\right|=\max_{\Theta_{n}\left(k\right)}\left|\underset{p\in P^{\prime }\left(k\right)}{⋃}\left\{p|I_{n,k}\left(p\right)=1\right\}\right|,$$

where $P^{\prime }\left(k\right)$ denotes the set of GPS points relevant for optimization at timeslot *k* (actual data for the oracle strategy, and previous timeslot data for the predictive strategy).

The computational complexity presents the primary challenge. With *M* beams and *L* discrete angle choices per beam (e.g., L=360 for $1^{\circ }$ resolution), the search space size is $O(L^{M})$. An exhaustive search would require evaluating approximately $L^{M}$ combinations per RSU per timeslot (e.g., $360^{3}\approx 46.7$ million evaluations for M=3), which is computationally prohibitive for real-time operation across a network. This intractability necessitates an efficient heuristic algorithm.

### 5.2. Preprocessing: Angular Histograms

To facilitate rapid angle optimization, we preprocess the spatio-temporal data into angular histograms for each RSU. For each deployed RSU $r\in A^{*}$, we compute a 3D tensor AngularHistograms[r,k,a]. This tensor stores the aggregated GPS count within RSU *r*’s communication range *R* during timeslot *k*, arriving from angular direction $a\in [0^{\circ },359^{\circ }]$ (with $1^{\circ }$ resolution).

This transformation effectively condenses the 2D spatial distribution of traffic around the RSU into a 1D angular distribution, drastically reducing the input dimensionality for the optimization algorithm. The computation involves iterating over all grid cells (i,j) within the RSU’s coverage radius, calculating the azimuth angle α(i,j) from the RSU center to the cell center, and accumulating the traffic count C[i,j,k] into the corresponding angular bin a=⌊α(i,j)⌋.

### 5.3. Sequential Angular Search (SAS) Algorithm

We propose the Sequential Angular Search (SAS) algorithm, an efficient greedy heuristic designed to solve the real-time beam angle optimization problem in Equation (11). The fundamental intuition is to sequentially select beam orientations that cover the densest angular sectors not yet covered by previously placed beams.

The SAS algorithm, detailed in Algorithm 2, operates as follows:**Algorithm 2** Sequential Angular Search (SAS)**Require:** Angular histogram *H* (length L=360), Number of beams *M*, Beamwidth *W* (in degrees)**Ensure:** Optimal beam center angles $\Theta =\{\theta_{1},\theta_{2},\ldots ,\theta_{M}\}$   1: Θ←∅▷ Initialize empty set of angles   2: $H_{\mathrm{current}}\leftarrow H.\mathrm{copy}\left(\right)$▷ Working copy of the histogram   3: **for** m=1 to *M* **do**   4:    $\left(\theta_{m},\mathrm{score}\right)\leftarrow \mathrm{FindBestBeam}\left(H_{\mathrm{current}},W\right)$   5:    **if** score<ϵ
**then**▷ Stop if no significant points remain   6:          **break**   7:    **end if**   8:    $\Theta .\mathrm{append}(\theta_{m})$   9:    $H_{\mathrm{current}}\leftarrow \mathrm{MaskHistogram}\left(H_{\mathrm{current}},\theta_{m},W\right)$▷ Remove covered sector 10: **end for** 11: **return** Θ

The algorithm relies on two core sub-procedures:

- FindBestBeam($H^{\prime }$, *W*): This function identifies the center angle $\theta^{*}$ of a sliding window of width *W* that maximizes the sum of counts in the current histogram $H^{\prime }$. It is implemented efficiently in O(L) time using a cumulative sum array on a circularly padded version of $H^{\prime }$, avoiding redundant calculations. The function returns the optimal angle $\theta^{*}$ and the corresponding coverage score.
- MaskHistogram($H^{\prime }$, $\theta^{*}$, *W*): This function returns a modified histogram where the counts in the angular range $\left[\theta^{*}-W/2,\theta^{*}+W/2\right]$ (handling modulo $360^{\circ }$ wrap-around) are set to zero. This prevents the algorithm from repeatedly selecting the same high-density region in subsequent iterations.

The overall complexity of SAS is O(M×L), making it highly suitable for real-time execution within short operational timeslots.

### 5.4. Comparative Strategy Definition

To rigorously evaluate the performance benefits of dynamic control and assess our predictive approach, we define three distinct operational strategies for the fast-timescale module:

1. Strategy A: Static-Optimal (Baseline): This strategy represents the best possible static configuration. The SAS algorithm is executed once for each RSU using the histogram aggregated over the entire historical period (e.g., one full day), $H_{\mathrm{total}}=\sum_{k=1}^{K}\mathrm{AngularHistograms}\left[r,k,:\right]$. The resulting fixed beam angles $\Theta_{\mathrm{static}}$ are applied uniformly across all timeslots. This provides a fundamental baseline against which dynamic strategies are compared.
2. Strategy B: Predictive-Dynamic (Proposed Practical Method): This strategy emulates a practical online system operating with a minimal delay. To determine the angles $\Theta_{\mathrm{predict}}\left(k\right)$ for the current timeslot *k*, the SAS algorithm is executed using the traffic data from the immediately preceding timeslot, $H_{k-1}=\mathrm{AngularHistograms}\left[r,k-1,:\right]$. This approach leverages the inherent short-term temporal correlation in urban traffic patterns.
3. Strategy C: Oracle-Dynamic (Theoretical Upper Bound): This strategy establishes the theoretical performance ceiling achievable by the SAS algorithm with perfect instantaneous information. For each timeslot *k*, SAS is executed using the actual histogram for that same timeslot, $H_{k}=\mathrm{AngularHistograms}\left[r,k,:\right]$, yielding $\Theta_{\mathrm{oracle}}\left(k\right)$. While unrealizable in practice, it quantifies the maximum potential gain from ideal dynamic control.

## 6. Performance Evaluation

This section details the experimental setup and presents the results obtained from evaluating our proposed dual-timescale framework using real-world and synthetic datasets.

### 6.1. Experimental Setup

#### 6.1.1. Datasets

We utilize two primary dataset types:

- Chengdu Didi Dataset: A large-scale GPS trajectory dataset collected from Didi Chuxing vehicles in Chengdu, China, during October 2016. The study focuses on the central urban area, defined by the longitude range of [104.0397°, 104.12705°] and the latitude range of [30.6554°, 30.73017°]. After cleaning and preprocessing, the dataset contains approximately 40 million GPS points per day.
- Synthetic Dataset: To test our framework under idealized conditions, we constructed a synthetic dataset. This was motivated by the limited performance gains on real data, potentially due to road network constraints. The synthetic data is designed to exhibit high spatial and temporal volatility, mimicking our theoretical high-potential scenarios.

It is worth noting that while the real-world dataset is from 2016, it remains a widely recognized benchmark in the fields of intelligent transportation and mobile computing [30,31,32]. Furthermore, the fundamental spatio-temporal characteristics of urban traffic, such as tidal flows driven by land-use patterns and road topology, exhibit long-term structural stability. Therefore, it is representative and effective to extract mobility patterns from the dataset as the basis for evaluating the algorithm’s performance.

All datasets are processed into the 3D spatio-temporal cube *C* as described in Section 3.

#### 6.1.2. Evaluation Metrics

The primary performance metric is the Total Traffic Coverage ($C_{total}$), calculated as the sum of GPS points covered by the entire network of *N* RSUs over a 24-h period (Equation (11)). We analyze the performance of the three control strategies (A, B, C) using the following derived metric: Maximum Potential Dynamic Gain ($G_{pot}$): $G_{pot}=C_{total}\left(C\right)-C_{total}\left(A\right)$.

#### 6.1.3. Parameter Settings

The default parameters employed throughout the simulations, unless varied for sensitivity analysis, are summarized in Table 1.

#### 6.1.4. Baselines

To evaluate the effectiveness of our DPS-based deployment, we compare it against the following baseline deployment algorithms, all run with N=32:

- Traffic-Greedy: A conventional greedy algorithm for static coverage that uses a heatmap based solely on the 24-h average traffic volume.
- I-RSU (Baseline): A classic intersection-based heuristic prioritizing deployment near major road intersections.
- Random (Baseline): This method randomly selects *N* candidate locations for deployment, serving as a lower-bound reference.

Each resulting deployment configuration is then evaluated using the A, B, and C control strategies.

### 6.2. Overall Performance Comparison

We first conduct a comprehensive comparison of network performance. This involves evaluating the four deployment strategies (DPS-Greedy, Traffic-Greedy, I-RSU, Random) using the three control strategies (Static-A, Predictive-B, Oracle-C).

**Figure 5:**
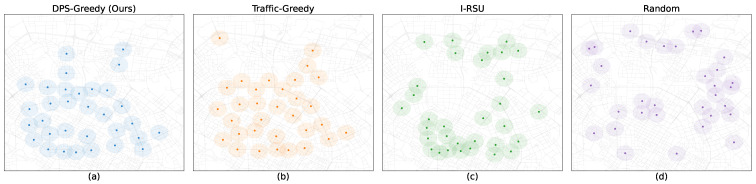
Deployment Visualization under Different Deployment Strategies (N=32) (axis units: decimal degrees).

Figure 5 visualizes the resulting physical placements for all four deployment strategies. Table 2 presents the core quantitative results. As shown in the table, **DyDO** (employing the DPS-Greedy strategy) consistently yields the highest total coverage ($C_{total}$) under all three control scenarios (A, B, and C). Notably, its Oracle-Dynamic (C) performance ($26.15\times 10^{6}$) and Potential Gain ($0.67\times 10^{6}$) are the highest, confirming that **DyDO** successfully identifies locations with the greatest true potential for dynamic adaptation. This outperforms the traditional Traffic-Greedy method, highlighting the inadequacy of optimizing for average volume.

**Figure 6:**
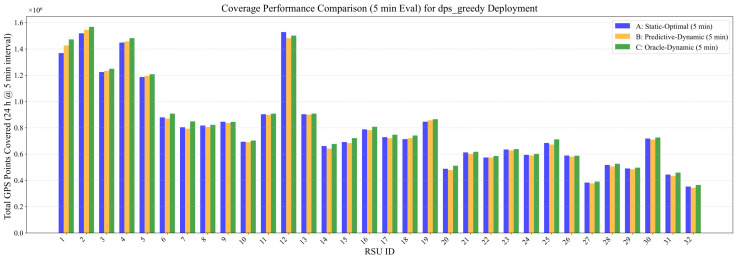
Coverage performance comparison: Static-Optimal (A) vs. Predictive-Dynamic (B) vs. Oracle-Dynamic (C) for each of the 32 DPS-deployed RSUs.

Figure 6 provides a detailed per-RSU breakdown of the coverage performance for the 32 RSUs deployed via our DPS-Greedy method. This chart directly compares the performance of the Static-Optimal (A), Predictive-Dynamic (B), and Oracle-Dynamic (C) control strategies for each individual RSU. Globally, Strategy C (Oracle) establishes the theoretical performance upper bound, consistently outperforming the Static-Optimal (A) baseline. Furthermore, the behavior of the Predictive-Dynamic (B) strategy, which uses data from timeslot k−1 to configure beams for the current timeslot *k*, merits discussion. This approach relies on the short-term temporal correlation of traffic patterns. However, when abrupt spatio-temporal shifts occur—making the traffic distribution at k−1 a poor predictor for *k*—this strategy can lead to suboptimal beam alignment. As observed in Figure 6 for some negative-gain RSUs (e.g., RSU #12, #25), this prediction error can occasionally cause Strategy B to underperform the stable Static-Optimal (A) baseline.

### 6.3. Case Study: Single RSU Beam Steering Snapshots

**Figure 7:**
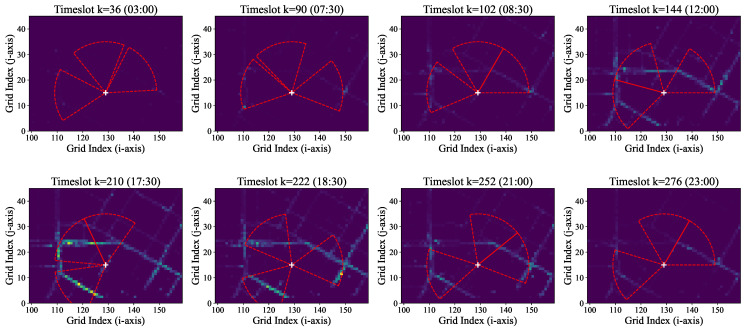
Single RSU beam steering snapshots for the highest-DPS RSU, showing dynamic adaptation to traffic at 8 different times of day (spatial coordinates are shown in grid indices, corresponding to the metric UTM projection described in Section 3).

To provide a qualitative understanding of the dynamic control module, we conduct a case study on a single RSU. We selected the first RSU deployed by our DPS-Greedy algorithm, which is the location with the highest Dynamic Potential Score (DPS). Figure 7 visualizes the beam steering behavior (using Predictive-Dynamic Strategy B) of this single RSU at eight representative snapshots throughout a 24-h period.

The visualization clearly demonstrates the RSU’s adaptability. During low-traffic periods (e.g., 03:00), the beams cover the few active vehicles. As the morning rush hour begins (e.g., 09:00), the beams re-orient to cover the dense, directional flow of traffic. This dynamic tracking of traffic hotspots, impossible for a static RSU, validates the necessity and efficacy of the fast-timescale SAS control algorithm.

### 6.4. Sensitivity Analysis

**Figure 8:**
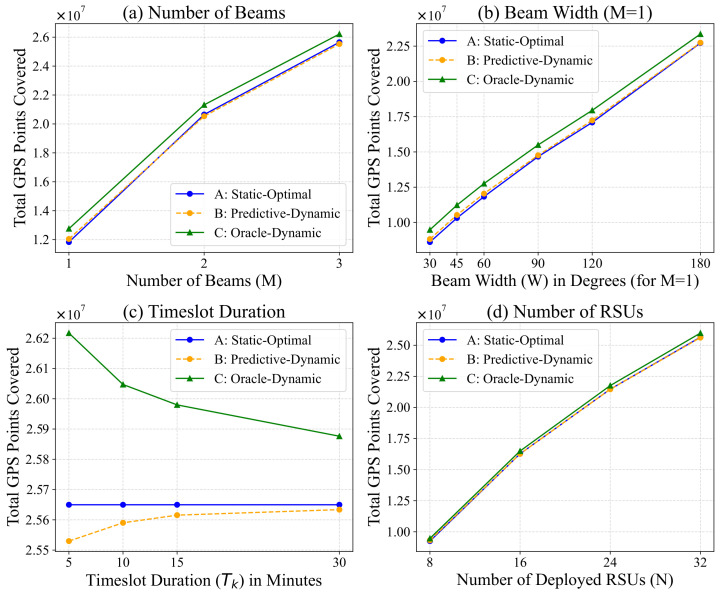
Sensitivity analysis of three control strategies (A–C) with respect to key parameters: (**a**) Number of Beams *M*, (**b**) Beamwidth *W*, (**c**) Timeslot Duration $T_{k}$, (**d**) Number of RSUs *N*.

We now analyze the framework’s sensitivity to four key parameters: number of beams (*M*), beamwidth (*W*), timeslot duration ($T_{k}$), and number of RSUs (*N*). We vary one parameter at a time while keeping others at their default values (see Table 1). The results are presented in Figure 8.

- Number of Beams (*M*) and Beamwidth (*W*): We tested M∈{1,2,3} and $W\in \{30^{\circ },45^{\circ },60^{\circ },90^{\circ },120^{\circ }\}$. As shown in Figure 8b,c, the performance improvement from M=1 to M=3 is clear, but variations in *M* (around 3) and *W* (around $60^{\circ }$) do not lead to significant performance changes. This suggests our framework is robust to these specific antenna hardware configurations, and our default choice of $M=3,W=60^{\circ }$ is effective.
- Number of RSUs (*N*): We tested N∈{8,16,24,32}. Figure 8a shows that as *N* increases, the performance of all strategies (A, B, and C) improves slightly. This is expected, as more RSUs cover more of the map’s high-potential areas.
- Timeslot Duration ($T_{k}$): This parameter, tested for $T_{k}\in \left\{5,10,15,30\right\}$ min, reveals the most interesting trade-off (Figure 8e). As the timeslot duration becomes longer, the performance of the Oracle-Dynamic (C) strategy decreases. This is because perfect information becomes less precise, as the angle choice is averaged over a longer, more varied period. Conversely, the performance of our Predictive-Dynamic (B) strategy shows a slight increase. This suggests that longer timeslots (e.g., 15–30 min) smooth out high-frequency traffic noise, making the traffic from the previous slot (k−1) a more stable and reliable predictor for the current slot (*k*).

### 6.5. Evaluation on Synthetic Dataset

We observed that the PotentialGain (C−A) on the real-world Chengdu dataset, while positive, is limited. We hypothesize this is due to the strong spatial constraints of the existing road network, which inherently limits the spatio-temporal volatility that 6DMA-RSUs can exploit.

**Figure 9:**
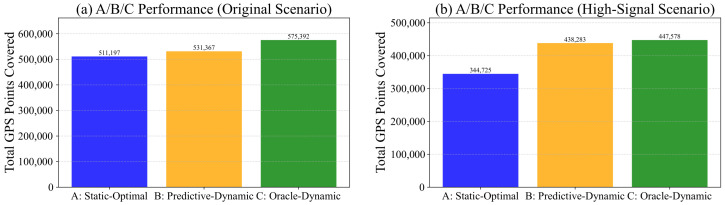
Synthetic evaluation comparison, showing a much larger performance gap between static (A) and dynamic (B, C) strategies, confirming the framework’s efficacy under ideal conditions.

To test our framework’s potential under more idealized conditions, we generated a synthetic dataset specifically designed to exhibit high spatial and temporal dynamics (e.g., strong, orthogonal tidal flows at an intersection). We then applied our deployment and optimization strategies. As shown in Figure 9, the results on this synthetic dataset were ideal. The performance gap between the dynamic strategies (B and C) and the static strategy (A) is larger than in the real-world data. This confirms that our DPS-Greedy deployment and SAS-based optimization framework are highly effective at capturing dynamic gains when deployed in environments that possess high intrinsic dynamic potential.

## 7. Conclusions

This paper introduced **DyDO**, a novel framework for the joint deployment and optimization of 6DMA-RSUs in V2X networks. To address the critical deployment–optimization mismatch, our framework decouples the problem into a slow-timescale strategic phase and a fast-timescale tactical phase. The core innovations are the Dynamic Potential Score (DPS) for identifying deployment locations with high dynamic adaptation potential, and the Sequential Angular Search (SAS) algorithm for efficient real-time beam steering. Extensive simulations on real-world data demonstrate that the proposed DyDO achieves a total traffic coverage of $26.15\times 10^{6}$ GPS points and a dynamic potential gain of $0.67\times 10^{6}$ points, which both significantly outperforms conventional static approaches. Furthermore, the proposed predictive-dynamic control using SAS captures the vast majority of the theoretical performance gains achievable through dynamic beam adaptation. It is important to acknowledge that practical implementation faces challenges, including the mechanical properties of antenna structures, the energy consumption associated with dynamic adjustments, and the acquisition and utilization of real-time traffic data. Despite these hurdles, this work establishes a foundational paradigm for designing and operating dynamic wireless infrastructure, paving the way for more efficient and adaptive 6G-V2X systems. Future work will incorporate QoS constraints and multi-objective optimization.

## Figures and Tables

**Table 1:** Default Simulation Parameter Settings.

Parameter	Symbol	Default Value
Deployment Budget	*N*	32 RSUs
RSU Coverage Radius	*R*	500 m
Number of Beams per RSU	*M*	3
Beamwidth	*W* or β	$60^{\circ }$
Grid Size	*G*	25 m
Timeslot Duration	$T_{k}$	5 min
Number of Timeslots	*K*	288 (24 h)
SAS Angular Resolution	*L*	360 ($1^{\circ }$)

**Table 2:** Network Performance ($C_{total}$) under Different Deployment Strategies (N=32).

Deployment Strategy	Static (A)/$\times 10^{6}$	Predictive (B)/$\times 10^{6}$	Oracle (C)/$\times 10^{6}$	Potential Gain (C−A)/$\times 10^{6}$
**DPS-Greedy (DyDO)**	**25.48**	**25.47**	**26.15**	**0.67**
Traffic-Greedy	24.15	23.96	24.68	0.53
I-RSU	19.24	19.05	19.63	0.39
Random	13.69	13.59	13.95	0.26

## Data Availability

The data presented in this study are available on request from the corresponding author.
